# Impact of Moral Distress, Person-Centred Care, and Nursing Professional Pride on Turnover Intention Among Intensive Care Unit Nurses in South Korea: A Cross-Sectional Study

**DOI:** 10.3390/healthcare14010022

**Published:** 2025-12-21

**Authors:** WonSuk Choi, Younjae Oh

**Affiliations:** 1 Graduate School of Nursing Science, Hallym University, Hallymdaehakgil 1, Chuncheon 24252, Republic of Korea; 2 Hallym University Sacred Heart Hospital, Gwanpyeong-ro 170beon-gil, Dongan-gu, Anyang-si 14068, Republic of Korea; 3 Research Institute of Nursing Science, College of Nursing, Hallym University, Hallymdaehakgil 1, Chuncheon 24252, Republic of Korea

**Keywords:** turnover intention, moral distress, person-centred care, nursing professional pride, nurses, intensive care unit, cross-sectional study

## Abstract

Background/Objectives: Turnover intention among intensive care unit (ICU) nurses remains consistently higher than that observed in other clinical departments. A weakened professional identity and exposure to ethically challenging situations may further intensify nurses’ intention to leave. This study aimed to examine the influence of moral distress, person-centred care, and nursing professional pride on turnover intention among ICU nurses in South Korea. Methods: A descriptive cross-sectional design was employed using a convenience sample of 203 ICU nurses from three general hospitals in South Korea. Data were obtained between 26 September and 31 October 2024 and analysed using IBM SPSS Statistics for Windows, version 29.0.2.0, with *t*-tests, one-way analysis of variance, Pearson’s correlation coefficients, and stepwise multiple regression analysis. Results: Two subdomains of nursing professional pride—role satisfaction and willingness to stay—along with gender (male) and the futile care subdomain of moral distress were the main factors influencing turnover intention. These variables collectively explained 24.9% of the variance in turnover intention (F = 17.78, *p* < 0.001). Conclusions: Strengthening nursing professional pride—particularly role satisfaction and willingness to stay—and reducing futile care-related moral distress may help lower ICU nurses’ turnover intention. Organisational strategies, including ethical management programmes and supportive policies, are recommended to enhance nursing professional pride, alleviate moral distress, and promote long-term nurse retention.

## 1. Introduction

A recent meta-analysis reported that the turnover intention among intensive care unit (ICU) nurses is 27.7%, markedly higher than that observed in other clinical departments [1]. High turnover in ICUs contributes to workforce shortages, intensifies nurses’ workloads [2], and induces secondary turnover among remaining staff [3]. These conditions undermine the professionalism and quality of nursing care and may adversely affect patient safety [4,5].

Turnover intention among ICU nurses arises from occupational factors—such as job stress, burnout, job satisfaction, and organisational commitment [1,6]—as well as ethical factors within the work environment, including ethical climate, ethical leadership, and moral distress [7,8]. Among these, moral distress has garnered increasing attention as a critical determinant of nurses’ psychological well-being and retention within rapidly evolving healthcare systems [9,10]. The growing complexity and unpredictability of ethical dilemmas in modern clinical practice intensifies moral distress, imposing additional psychological and ethical burdens on nurses [11,12].

Moral distress has been conceptualised following Jameton’s [13] and Wilkinson’s [14] research, as well as subsequent analyses by McCarthy and Deady [15], which distinguish it from moral uncertainty and general emotional distress. In this framework, moral distress arises when nurses are certain of the ethically appropriate course of action but are constrained from acting on it by institutional, hierarchical, or contextual barriers [13]. Although experiences of moral distress are typically accompanied by strong emotions (e.g., frustration, guilt, and anxiety), they cannot be reduced to emotional dysregulation or individual coping failure; rather, they stem from ethical conflict and externally imposed constraints within the care environment [11,16]. Organisational and situational constraints that prevent nurses from delivering person-centred or dignified care can exacerbate moral distress and create additional ethical challenges [11,17]. International studies indicate that regulatory ambiguity, unclear legal guidance, and ethical–legal dilemmas can heighten moral distress by limiting nurses’ professional autonomy and generating value conflicts [18,19]. Moral distress is widely reported across diverse healthcare systems, highlighting it as a global phenomenon shaped by the ethical and regulatory contexts in which nurses practise [19,20].

Patients in ICUs frequently cannot communicate or advocate for their preferences due to critical illness or physical incapacity. Consequently, ICU nurses face ethically challenging situations such as providing life-sustaining treatment to terminally ill patients, witnessing treatment delays due to limited resources or questionable professional conduct [17,21], and applying physical restraints to prevent self-harm or interference with treatment [22,23]. ICU nurses are at increased risk of experiencing moral distress when patients’ autonomy and dignity are inadequately protected, potentially undermining their moral integrity [17,24]. In response, some nurses may withdraw from close engagement with patients, reduce advocacy, or become morally desensitised to avoid repeated ethically distressing encounters [11,16]. These experiences can evoke guilt and accumulate over time as moral residue [16], intensifying subsequent moral distress and contributing to psychological strain, professional disillusionment, and intentions to leave, ultimately undermining care quality and patient outcomes [10,12].

The ICU is also a highly technological environment where life-sustaining interventions rely on advanced devices and monitoring systems. While essential, this technology can obscure the interpersonal and existential dimensions of care, prompting nurses to reflect on human existence, personhood, and dignity [11,17]. Therefore, person-centred care has increasingly been recognised as a core paradigm in critical care nursing, affirming each patient’s inherent worth and dignity while providing holistic care tailored to individual needs and preferences [17,25]. ICU patients value nurses’ empathy, emotional support, and respect for dignity in addition to professional competence and continuous bedside presence, which foster safety and trust [26,27]. However, communication challenges within multidisciplinary teams, heavy workload, technological demands, and high stress levels usually hinder person-centred care delivery [28,29]. Continuous reliance on equipment and monitors may further constrain nurse–patient interaction and risk reducing patients to mere objects of treatment [11,30]. Such conditions contribute to secondary traumatic stress and burnout, which consequently impede person-centred practise and heighten turnover intention [11,31].

Nursing professional pride—a construct encompassing a nurse’s sense of calling, confidence, and responsibility towards the profession—has emerged as a significant predictor of retention, alongside moral and relational factors [32]. Nurses who demonstrate a strong professional identity, trustworthiness, and ethical responsibility are better equipped to deliver holistic, high-quality care [11,33]. A well-established professional identity enhances self-esteem and confidence, thereby fostering greater job satisfaction, organisational commitment, and professional performance [34]. Higher levels of nursing professional pride are consistently associated with nurses’ intention to remain in the profession and improved nursing performance [35,36]. Nurses who experience autonomy and self-fulfilment tend to report greater nursing professional pride, reinforcing their motivation to remain in the workforce [32,37]. However, South Korean nurses are frequently faced with limited professional autonomy, passive professional roles, and restricted decision-making authority [17,38]. Reduced nursing professional pride arising from low self-esteem, constrained autonomy, and job dissatisfaction may contribute to increased turnover intention among ICU nurses [34,39].

These findings collectively suggest that morally constraining conditions that generate moral distress, impede person-centred care, and weaken nursing professional pride can gradually erode ICU nurses’ sense of meaning, professional identity, and commitment. This erosion may manifest as psychological strain, disengagement from the nursing role, and ultimately, increased turnover intention. Therefore, the present study aimed to examine the influence of moral distress, person-centred care, and nursing professional pride on turnover intention among ICU nurses in South Korea.

## 2. Materials and Methods

### 2.1. Participants and Settings

This study employed a cross-sectional descriptive design. A convenience sample of 203 ICU nurses was recruited from three general hospitals, each with more than 200 beds, located in two major regions of South Korea: Seoul and Gyeonggi Province. Based on previous research, novice nurses with <6 months of clinical experience, who were still undergoing orientation and training and had not yet begun performing independent nursing duties, were excluded [11]. Unit managers, whose roles and work environments differ from those of staff nurses, were also excluded [40,41]. The required sample size was calculated using G*Power (version 3.1.9.7) to ensure a significance level of α = 0.05, with 90% power and a medium effect size of 0.15. Considering a 15% allowance for potential dropouts and errors, the required total sample size was calculated to be 240.

Data collection occurred between 26 September and 31 October 2024. Participants completed self-administered questionnaires, sealed them in envelopes to ensure confidentiality, and returned them to the researcher during hospital visits. Of the 240 questionnaires distributed, 232 were returned (response rate = 96.7%). Ultimately, 203 questionnaires were retained for the final analysis after excluding incomplete or unclear responses.

### 2.2. Instruments

#### 2.2.1. General Characteristics

The questionnaire included items assessing the participants’ general characteristics, categorised into seven variables: age, gender, marital status, educational level, religion, total years of nursing experience, and years of experience working in the ICU.

#### 2.2.2. Turnover Intention

Turnover intention was assessed using the Korean version of the Turnover Intention Scale (KTI), originally developed by Lawler [42] and subsequently validated for nurses by Park [43]. Permission to use the Korean version was obtained from the author. The revised KTI comprises four items rated on a 5-point Likert scale ranging from 1 (none) to 5 (severe), with higher scores indicating stronger turnover intention. Lawler [42] and Park [43] reported Cronbach’s alpha coefficients of 0.83 and 0.88, respectively. The internal consistency reliability of the KTI in the present study was 0.85.

#### 2.2.3. Moral Distress

Moral distress was measured using the Korean version of the Moral Distress Scale–Revised (KMDS-R), validated by Chae et al. [44] based on the original Moral Distress Scale–Revised (MDS-R) developed by Hamric et al. [45]. Permission to use the KMDS-R was obtained from the authors. The KMDS-R comprises 21 items assessing situations that cause moral distress across the following five subdomains: futile care (five items), nursing practice (five items), institutional and contextual factors (four items), physician practice (four items), and limitations in raising ethical issues (three items). Each item is rated on two separate 5-point Likert scales measuring the frequency (0 = never to 4 = very frequently) and intensity (0 = none to 4 = great extent) of moral distress. Specifically, the total moral distress score is calculated by multiplying the frequency and intensity score for each item, with a value of 0 assigned when neither frequency nor intensity are reported. The overall score is obtained by summing the results across all items, with higher scores indicating greater moral distress. Hamric et al. [45] and Chae et al. [44] reported Cronbach’s alpha coefficients of 0.89 and 0.91, respectively. The internal consistency reliability of the KMDS-R in the present study was 0.91.

#### 2.2.4. Person-Centred Care

Person-centred care was assessed using the Korean version of the Person-Centred Practice Inventory–Staff (K-PCPI-S), validated by Kim and Tak [46] based on the original Person-Centred Practice Inventory–Staff developed by Slater et al. [47]. Permission to use the K-PCPI-S was obtained from the authors. The instrument comprises 51 items measuring perceptions of person-centred attitudes and behaviours across the following three subdomains: prerequisites (18 items), care environment (25 items), and person-centred processes (8 items). Each item is rated on a 5-point Likert scale ranging from 1 (strongly disagree) to 5 (strongly agree), with higher scores reflecting higher levels of person-centred care. Kim and Tak [46] reported a Cronbach’s alpha coefficient of 0.95. In the present study, the internal consistency reliability was also 0.95, confirming the scale’s reliability.

#### 2.2.5. Nursing Professional Pride

Nursing professional pride was measured using the Nursing Professional Pride (NPP) scale, developed and validated by Jeon et al. [48]. Permission to use the NPP was obtained from the authors. The instrument includes 21 items categorised into five dimensions: feeling of vocation (six items), role satisfaction (six items), role as a problem solver (six items), self-achievement (four items), and willingness to stay (five items). Items are rated on a 5-point Likert scale ranging from 1 (not at all) to 5 (very high), with higher scores indicating higher levels of nursing professional pride. Jeon et al. [48] reported a Cronbach’s alpha coefficient of 0.96. The internal consistency reliability of the NPP in the present study was 0.90.

### 2.3. Data Analysis

Data were analysed using Cronbach’s alpha reliability analysis, *t*-tests, one-way analysis of variance with post hoc Scheffé tests, Pearson’s correlation coefficients, and stepwise multiple regression analysis. All analyses were performed using IBM SPSS Statistics for Windows, version 29.0.2.0 (IBM Corp., Armonk, NY, USA).

Stepwise multiple linear regression was conducted to identify predictors of turnover intention. Given the partly exploratory nature of this study and the inclusion of several conceptually related psychosocial and ethical variables (including subscales of moral distress and nursing professional pride), stepwise selection was used to derive a parsimonious model and reduce redundancy among correlated predictors. All candidate variables that showed significant bivariate associations with turnover intention were entered into the stepwise procedure. Variables were entered or removed according to the default probability criteria in SPSS (entry *p* < 0.05, removal *p* > 0.10). Assumptions of multiple linear regression were examined prior to the final model interpretation. Normality, linearity, and homoscedasticity were assessed using residual and normal probability plots, which indicated no serious violations. Influential cases were evaluated using Cook’s distance, and no observations exceeded the conventional threshold.

### 2.4. Ethical Considerations

This study was conducted in accordance with the Declaration of Helsinki and was approved by the Institutional Review Board of Hallym University before data collection (approval no. HIRB-2024-070). All participants provided written informed consent after being informed of the study’s purpose, confidentiality measures, and assurance of anonymity. Participation was voluntary, and participants were informed that they could withdraw from the study at any time without repercussions. They were also assured that their responses would be used solely for research purposes, would not be shared with hospital management, and would be reported only in aggregated form. No monetary incentives were provided. Participants who were unable to return the questionnaire directly to the researcher due to work shifts were instructed to seal it in an envelope to maintain confidentiality.

## 3. Results

### 3.1. Differences in Turnover Intention Based on General Characteristics

Turnover intention differed significantly according to gender and years of experience working in the ICU. Female nurses reported significantly higher turnover intention than male nurses (t = −4.364, *p* < 0.001). Nurses with >2 years of experience working in the ICU showed significantly higher turnover intention than others (F = 6.62, *p* < 0.001) (Table 1).

### 3.2. Levels of Turnover Intention, Moral Distress, Person-Centred Care, and Nursing Professional Pride

The mean scores for turnover intention and moral distress were 3.49 (standard deviation [SD] = 0.80) and 90.45 (SD = 52.56), respectively. Among the five subdomains of moral distress, futile care (mean = 6.20, SD = 3.46) and physician practice (mean = 2.46, SD = 2.50) displayed the highest and lowest mean scores, respectively.

For person-centred care, the mean score was 177.99 (SD = 22.39). Among its three subdomains, person-centred processes (mean = 3.65, SD = 0.50) and care environment (mean = 3.29, SD = 0.54) displayed the highest and lowest mean scores, respectively.

The mean score for nursing professional pride was 98.05 (SD = 13.57). Among its five subdomains, role as a problem solver (mean = 4.11, SD = 0.52) and role satisfaction (mean = 2.87, SD = 0.61) displayed the highest and lowest mean scores, respectively (Table 2).

### 3.3. Correlations Between Turnover Intention, Moral Distress, Person-Centred Care, and Nursing Professional Pride

Turnover intention was positively correlated with moral distress (r = 0.293, *p* < 0.001) and negatively correlated with person-centred care (r = −0.189, *p* = 0.007) and nursing professional pride (r = −0.359, *p* < 0.001).

All moral distress subdomains were significantly correlated with turnover intention. Within the person-centred care subdomains, only one, Care environment (r = −0.217, *p* = 0.002) showed a significant relationship with turnover intention. All nursing professional pride subdomains were significantly correlated with turnover intention (Table 3).

### 3.4. Impact of Moral Distress, Person-Centred Care, and Nursing Professional Pride on Turnover Intention

Stepwise multiple regression analysis identified four significant predictors of turnover intention: the futile care subdomain of moral distress, the role satisfaction and willingness-to-stay subdomains of nursing professional pride, and gender. These variables explained 24.9% of the variance in turnover intention (F = 17.78, *p* < 0.001) (Table 4). Higher levels of moral distress related to futile care and being male were associated with increased turnover intention, whereas higher role satisfaction and willingness to stay were associated with lower turnover intention. In the final model, the standardized regression coefficients were as follows: role satisfaction (*β* = −0.261, *p* < 0.001), willingness to stay (*β* = −0.193, *p* = 0.004), gender (male; *β* = −0.225, *p* < 0.001), and futile care (*β* = 0.168, *p* = 0.011).

The regression model was tested for multicollinearity and met statistical assumptions. The Durbin–Watson statistic was 1.894, indicating no autocorrelation of residuals. Furthermore, variance inflation factor values ranged from 1.075 to 1.221, well below the threshold of 10, confirming the absence of multicollinearity [49] (Table 4).

## 4. Discussion

This study examined the influence of moral distress, person-centred care, and nursing professional pride on turnover intention among ICU nurses in South Korea. Gender, moral distress related to futile care, and the role satisfaction and willingness to stay subdomains of nursing professional pride emerged as significant predictors of turnover intention, collectively explaining 24.9% of the variance.

The finding that the role satisfaction subdomain of nursing professional pride significantly reduced turnover intention indicates that satisfaction with one’s professional role is associated with a lower intention to leave. Clearly defined responsibilities and a strong sense of contribution are essential for maintaining motivation and professional pride in ICU settings, where nurses must continuously coordinate complex tasks, manage life-sustaining technologies, and collaborate within interdisciplinary teams [17,34]. A comparison of this finding with those of previous studies is difficult since only a few studies have examined the direct relationship between professional pride and turnover intention among ICU nurses.

Nevertheless, our findings align with evidence indicating that role-related perceptions are critical determinants of nurse retention. Role conflict and ambiguity increase turnover intention in high-stress environments [50,51], whereas role clarity and satisfaction enhance nurses’ intention to stay [52,53]. Furthermore, professional fulfilment and organisational commitment are strengthened when nurses understand their responsibilities, receive adequate supervisory support, and perceive fairness in workload allocation, which consequently lowers turnover intention [54,55]. These consistencies suggest that when nurses clearly understand and are satisfied with their professional roles, their sense of professional pride and organisational commitment is strengthened, ultimately lowering turnover intention.

The willingness to stay subdomain also significantly reduced turnover intention. In the original NPP scale, this subdomain reflects nurses’ willingness to remain in their current job, based on the perception that their role offers career stability and meaningful influence within the organisation [48]. This supports previous research showing that nurses are more likely to stay when they believe their work is valuable and their contributions are recognised within the team [52,56]. Yeşilyurt et al. [57] demonstrated that job motivation mediates the relationship between career barriers and turnover intention, suggesting that the impact of organisational constraints is attenuated when nurses are internally motivated by moral purpose, professional growth, and the meaningfulness of their work. Similarly, Pahlevan Sharif et al. [58] reported that external rewards such as pay or benefits are insufficient to sustain retention when deeper professional meaning is undermined. Furthermore, a strong willingness to stay may represent not only job satisfaction but also ethical commitment to patients and colleagues in the intensive care context, where nurses perform highly specialised and interdependent roles [17,27]. When organisations foster a supportive environment that validates nurses’ professional competence and significance, their willingness to remain in their role is strengthened, thereby reducing turnover intention [8].

Moral distress related to futile care significantly increased turnover intention. This supports previous findings indicating that repeated exposure to ethically distressing situations—such as providing treatment perceived as non-beneficial—intensifies emotional exhaustion and fosters intentions to leave [59,60]. Such moral distress can undermine nurses’ sense of professional meaning and competence, particularly when they feel powerless to advocate for patients’ dignity [10,61]. In Vieira et al.’s [61] scoping review on perceptions of futile care among ICU nurses, continuation of life-sustaining treatment with no expected benefit is frequently perceived as therapeutic futility, generating substantial moral conflict, and emotional exhaustion. Tension between the nurses’ professional conscience and institutional demands gives rise to moral distress when nurses are required to perform interventions they regard as non-beneficial or prolonging suffering [31,59]. These situations erode their sense of professional meaning and moral integrity, particularly when they are excluded from decision-making processes or lack the authority to advocate for futile treatment withdrawal [11].

Oh and Gastmans’s [11] systematic reviews on ethical issues in ICUs interpret moral distress not merely as psychological discomfort but as an ethical response to violations of moral integrity and ethical responsibility. When organisational structures suppress nurses’ moral agency and fail to recognise professional judgement, turnover can be understood not only as occupational disengagement but also as an ethical response to moral disintegration within the healthcare system. In South Korea, ICU nurses are usually excluded from decision-making related to futile care because of medical paternalism and hierarchical organisational structures, which are significant factors influencing nurses’ intention to change or leave their jobs [16,17]. Medical paternalism in healthcare systems with similar cultural or hierarchical characteristics has been shown to heighten moral distress and weaken nurses’ professional commitment, as reported in studies from Taiwan [62], Iran [63], and China [64].

Consistent with previous studies, the present findings confirm that moral distress related to futile care is a key predictor of turnover intention among ICU nurses. Nurses’ professional identity may be weakened when they are repeatedly constrained from providing care that aligns with their ethical judgement, increasing the risk of withdrawal and eventual turnover [10,17]. Therefore, organisational efforts to minimise futile treatment decisions, strengthen ethical communication, and promote shared decision-making within healthcare teams are essential. Concurrently, individual psychological resources such as emotion regulation [65,66] and psychological flexibility [67] may help nurses cope with ethically distressing situations, particularly when understood as part of cultivating moral resilience—that is, the capacity to sustain or restore integrity in the face of moral adversity [68]. Future research should examine how such personal resources and organisational conditions interact to alleviate moral distress and reduce turnover intention. Furthermore, workforce loss and economic impact were not assessed since the MDS-R is designed to measure moral distress, and this study examined turnover intention rather than actual turnover or organisational costs. These outcomes should be addressed in subsequent studies.

Gender also emerged as a significant predictor, with male nurses reporting lower turnover intention than female nurses. This finding is broadly consistent with studies suggesting that female nurses usually experience greater emotional burden, work–life imbalance, and limited authority compared with male nurses [16,36]. Female nurses may be more exposed to emotional exhaustion due to disproportionate expectations of emotional labour in interactions with patients and families, which can heighten moral distress and increase turnover intention [50]. Meanwhile, male nurses may perceive greater career mobility or leadership opportunities in ICU settings, potentially enhancing job satisfaction and reducing turnover intention [36]. In collectivist and hierarchical healthcare systems such as that in South Korea, gender norms may also reinforce unequal communication patterns and restrict female nurses’ ability to assert professional opinions, further intensifying moral distress [17,38]. Nonetheless, this gender difference should be interpreted with caution. The proportion of male nurses in the sample was relatively small, and alternative explanations—such as differences in role expectations, career trajectories, and cultural norms regarding gender and work in South Korea—may have also contributed to the observed pattern [9,38]. Therefore, targeted organisational strategies that address gender-specific stressors, including flexible scheduling, psychosocial support and attention to unequal communication, and decision-making dynamics, may be important for improving workforce stability. However, some studies report no significant impact of gender on turnover intention [9]; such inconsistencies may reflect variations in cultural or organisational contexts.

## 5. Implications and Limitations

The findings of this study have meaningful implications for nursing practice and management. Reinforcing nursing professional pride and reducing moral distress are critical strategies for decreasing turnover intention among ICU nurses. These findings highlight that turnover intention among ICU nurses is not merely a staffing issue, but a concern rooted in professional identity, moral agency, and the dignity of care.

Despite the valuable insights and contributions of this study, some limitations exist. First, although data were collected from three hospitals across different regions, the use of convenience sampling may limit the generalisability of the findings. Second, the predominance of female respondents reflects the gender composition of the Korean nursing workforce, where male nurses accounted for only 4.8% of registered nurses in 2020 [69]. This imbalance may restrict understanding of gender-related differences. Future research should explore the relationships among nursing professional pride, moral distress, person-centred care, and turnover intention of male nurses, applying frameworks such as the Sex and Gender Equity in Research guidelines [70]. Third, the findings must be viewed considering South Korea’s hierarchical, bureaucratic, and protocol-driven hospital culture, which shapes ICU nurses’ experiences of moral distress and nursing professional pride. These contextual factors may also limit the results’ transferability to healthcare systems with different organisational structures or cultural expectations. Comparative research across countries is needed to determine whether these patterns are context-specific or generalisable. Fourth, turnover intention is influenced by multiple organisational, economic, and personal factors not captured in this study. Future research should incorporate a broader set of variables to more fully explain nurses’ intentions to leave. Fifth, additional factors at the level of personal psychological resources—such as psychological flexibility, emotion regulation, and moral resilience—may further illuminate pathways from morally constrained practise to turnover intention and warrant investigation in future research. Finally, stepwise multiple regression was used to identify the most influential predictors among several correlated psychosocial and ethical variables. However, since stepwise procedures may be susceptible to model instability and overfitting, the pattern of predictors observed in this study should be interpreted with caution.

## 6. Conclusions

This study examined the effects of moral distress, person-centred care, and nursing professional pride on turnover intention among ICU nurses. Reduced nursing professional pride—particularly lower role satisfaction and willingness to stay—and moral distress related to futile care were significant predictors of higher turnover intention. These findings indicate that ethical and psychosocial conditions in the ICU play a critical role in nurses’ decisions to remain in or leave their positions.

To address these issues, organisations should actively support nurses in preserving their moral integrity, recognise their professional contributions, and create conditions conducive to ethical practise. Concrete strategies include establishing structured ethical communication channels (e.g., ethics rounds, debriefings, and interprofessional case discussions), promoting shared decision-making in end-of-life care, and implementing programmes that acknowledge and reinforce nursing professional pride and role satisfaction. Interventions that foster individual psychological resources—such as emotion regulation, psychological flexibility, and moral resilience—may complement these organisational efforts and help alleviate moral distress. Together, such approaches may reduce turnover intention and contribute to a more stable and sustainable ICU nursing workforce.

## Figures and Tables

**Table 1:** Differences in turnover intention based on general characteristics (*n* = 203).

Characteristics	Categories	*n* (%) or Mean (SD)		Turnover Intention			
				Mean	(SD)	t of F	*p*
						Post Hoc Scheffé Tests	
Gender	Female	161	(79.3)	3.61	(0.78)	−4.36	<0.001
	Male	42	(20.7)	3.03	(0.73)		
Age (years)	20–25	54	(26.6)	3.42	(0.79)	1.19	0.313
	26–30	96	(47.3)	3.57	(0.80)		
	31–35	37	(18.2)	3.57	(0.79)		
	≥35	16	(7.9)	3.20	(0.92)		
		29.1	(5.7)				
Marital status	Married	32	(15.8)	3.57	(0.79)	0.20	0.656
	Unmarried	171	(84.2)	3.48	(0.80)		
Religion	With a religion	46	(22.7)	3.27	(0.95)	−1.92	0.059
	Without a religion	157	(77.3)	3.56	(0.74)		
Educational level	Associate degree	21	(10.3)	3.61	(0.68)	0.75	0.522
	Bachelor’s degree	159	(78.3)	3.51	(0.79)		
	Graduate degree	23	(11.4)	3.35	(1.06)		
Total years of nursing experience	<3	91	(44.8)	3.39	(0.77)	2.56	0.080
	3 to <8	65	(32.0)	3.68	(0.78)		
	≥8	47	(23.2)	3.45	(0.87)		
		5.9	(6.2)				
Years of experience working in the intensive care unit	<2 ^a^	52	(25.6)	3.12	(0.83)	6.62	<0.001
	2 to <4 ^b^	74	(36.5)	3.73	(0.69)	a < b	
	4 to <8 ^c^	45	(22.1)	3.46	(0.85)		
	≥8 ^d^	32	(15.8)	3.59	(0.73)		
		4.6	(4.3)				

^a, b, c^ and ^d^ groups from post hoc Scheffé tests. SD, standard deviation.

**Table 2:** Levels of turnover intention, moral distress, person-centred care, and nursing professional pride (*n* = 203).

Variables	Mean (SD)	Range	Min–Max
Turnover intention	3.49 (0.80)	1–5	1.00–5.00
Moral distress	90.45 (52.56)	0–336	0.00–300.00
Futile care	6.20 (3.46)		
Nursing practice	5.59 (3.61)		
Institutional and contextual factors	3.05 (2.30)		
Limitations in raising ethical issues	2.58 (2.59)		
Physician practice	2.46 (2.50)		
Person-centred care	177.99 (22.39)	51–225	121.00–225.00
Person-centred processes	3.65 (0.50)		
Prerequisites	3.62 (0.41)		
Care environment	3.29 (0.54)		
Nursing professional pride	98.05 (13.57)	27–135	50.00–135.00
Role as a problem solver	4.11 (0.52)		
Willingness to stay	3.96 (0.60)		
Self-achievement	3.72 (0.86)		
Feeling of vocation	3.56 (0.61)		
Role satisfaction	2.87 (0.72)		

SD, standard deviation; Min, minimum; Max, maximum.

**Table 3:** Correlations between turnover intention, moral distress, person-centred care, and nursing professional pride (*n* = 203).

		Turnover Intention	
		r	*p*
Moral distress		0.293	<0.001
Nursing practice		0.287	<0.001
Futile care		0.269	<0.001
Institutional and contextual factors		0.220	0.002
Limitations in raising ethical issues		0.207	0.003
Physician practice		0.139	0.048
Person-centred care		−0.189	0.007
Care environment		−0.217	0.002
Person-centred processes		−0.133	0.590
Prerequisites		−0.103	0.142
Nursing professional pride		−0.359	<0.001
Role satisfaction		−0.391	<0.001
Feelings of vocation		−0.311	<0.001
Willingness to stay		−0.260	<0.001
Self-achievement		−0.228	0.001
Role as a problem solver		−0.142	0.043

**Table 4:** Impact of moral distress, person-centred care, and nursing professional pride on turnover intention (*n* = 203).

Independent Variables	Unstandardised Coefficients		Standardised Coefficients			Collinearity Statistics	
	B	SE	*β*	t	*p*	Tolerance	VIF
(constant)	20.783	1.406					
Nursing professional pride							
Role satisfaction	−1.159	0.299	−0.261	−3.881	<0.001	0.819	1.221
Willingness to stay	−1.024	0.348	−0.193	−2.944	0.004	0.862	1.161
Gender *							
Male	−1.788	0.502	−0.225	−3.564	<0.001	0.931	1.075
Moral distress							
Futile care	0.031	0.012	0.168	2.580	0.011	0.871	1.147

* Reference group (coded as 0) for the dummy variable: gender (female); F = 17.78, *p* < 0.001, *R*^2^ = 0.264, Adjusted *R*^2^ = 0.249, *d*(*d_u_*) = 1.894; VIF, variance inflation factor; SE, standard error.

## Data Availability

Data can be made available upon request from the corresponding author of this study.
